# 

**Published:** 1898-11

**Authors:** 


### CONTENTS.

ORIGINAL COMMUNICATIONS—

Amputation of Breast: Tumor of Orbit: Supernumerary Digit: Gunshot Wound of Ankle. By Wm. Simpson Elkin, M.D., Atlanta, Ga. .......... 577

Medical Reminiscences of Forty Years Ago. By G. G. Roy, M.D., Atlanta, Ga.. 582

The Value of Iridectomy in Blindness, Resulting from Opaque Cornea. By A. Bethune Patterson, M.D., Atlanta, Ga .......... 585

History of the Medical Examining Board of Georgia. By E. R. Anthony, M.D., Griffin, Ga .......... 589

Cancer Viewed and Treated from the Standpoint of the General Practitioner, with Reports of Cases. By Bittle C. Keister, A.M., M.D., South Boston, Va .......... 592

SOCIETY REPORTS—

Chicago Gynecological Society .......... 603

CORRESPONDENCE—

The Progress of Medical Education .......... 610

Regular Board of Examiners .......... 613

EDITORIAL—

Do Physicians Know too Much .......... 616

The Quarantine Question .......... 618

The Georgia Medical Association .......... 620

NEWS AND NOTES .......... 623

BOOK REVIEWS, PAMPHLETS, EXCHANGES—

Book Reviews .......... 632

Reprints Received .......... 635

Magazines and Exchanges .......... 636

Magazine Notes .......... 637

SELECTIONS AND ABSTRACTS .......... 638

MEDICAL ITEMS—Continued .......... x, xi. xii, xiii, xiv, xv

ADVERTISERS' INDEX TO ADVERTISEMENTS .......... xvi

CLASSIFIED INDEX TO ADVERTISEMENTS .......... xvii

